# Supplementary material for: Chromothripsis during telomere crisis is independent of NHEJ, and consistent with a replicative origin
Source: Genome Res. 2019 May;29(5):737–49. doi: 10.1101/gr.240705.118 (PMC6499312; doi:10.1101/gr.240705.118)
Supplement: Supplemental Material [file supp_gr.240705.118_Supplemental_file_1.zip › contigs/annotated_contigs/DB48/contig.2.DB48_length_720_mean_cov_9.69166666667.docx]

**DB48_length_720_mean_cov_9.69166666667**

```
GAATTCTACCAGAGGTACAAGGAGGAGCTGGTACCATTCCTTCTGAAACTATTCCAATCAATAGAAAAAGAGGGAATCTTCCCTAACTC
 >chr9:88820248-88820625 + E=5e-215
ATTTTATGAGGCCAGCATCATCCTGATACCAAAGGCTGGCAGAGACACAACAAAAAAAGAGAATTTTAGACCAATATCCTTGATGAACA

TCGATGTGAAAATCCTCAATAAAATACTGGCAAACCAAATCCAGCAGCACATCAAAAAGCTTATCCATCACGATCAAGTGGGCTTCATC

CCTGGGATGCAAGGCTAGTTCAACATACACAAACCAATAAACATAATCCATCACATAAACCGAACCAAAGACAAAAACCACATGATTAT

CTCAATAGATGCAGAAAAGG|C|TTTATTCTGATATTGGTAGATTCTACTGAAGGCTTGTATGCAGGGACATGATGGGATCAAGTCAAA
                     >chr9:88639325-88639669 + E=4e-195
TCTGCTTGCCTGAGAATGGTTGAAAAGGGAGGCAGGGAAGCCAGTCACGTGAGAGAAAATGTTGGTCTTAACAAAGGTAGTGGCAGAGC

AGGTGGAAGAAAGTGGATGAAATCAAATGTTTTCAGAGGTACAGCTAATGGGACATGGTGAATGAGAGTTTCTGCCAAAATCAAGGATG

ATTTTTCAGAAATTTTTTTGGCTTTGGCAACTAGACGATGGTGGTGCCACTTGTGTAGATATCTAGGCAAGGTAGGAGACCAGGTTTGG

GATAAAGATT
```
